# Active Compounds with Medicinal Potential Found in Maxillariinae Benth. (Orchidaceae Juss.) Representatives—A Review

**DOI:** 10.3390/ijms24010739

**Published:** 2023-01-01

**Authors:** Monika M. Lipińska, Łukasz P. Haliński, Marek Gołębiowski, Agnieszka K. Kowalkowska

**Affiliations:** 1 Department of Plant Taxonomy and Nature Conservation, Faculty of Biology, University of Gdańsk, Wita Stwosza 59, 80-308 Gdansk, Poland; 2 Foundation Polish Orchid Association, 81-825 Sopot, Poland; 3 Laboratory of Analysis of Natural Compounds, Department of Environmental Analytics, Faculty of Chemistry, University of Gdańsk, Wita Stwosza 63, 80-308 Gdansk, Poland; 4 Department of Plant Cytology and Embryology, Faculty of Biology, University of Gdańsk, Wita Stwosza 59, 80-308 Gdansk, Poland

**Keywords:** active compounds, ethnobotany, medicine, orchids, phytochemistry

## Abstract

Orchids are widely used in traditional medicine for the treatment of a whole range of different health conditions, and representatives of the Neotropical subtribe Maxillariinae are not an exception. They are utilized, for instance, for their spasmolytic and anti-inflammatory activities. In this work, we analyze the literature concerning the chemical composition of the plant extracts and secretions of this subtribe’s representatives published between 1991 and 2022. Maxillariinae is one of the biggest taxa within the orchid family; however, to date, only 19 species have been investigated in this regard and, as we report, they produce 62 semiochemicals of medical potential. The presented review is the first summary of biologically active compounds found in Maxillariinae.

## 1. Introduction

Subtribe Maxillariinae Benth. counting ca. 420 [1] to 750 taxa [2] is one of the richest species groups within the orchid family. It is also one of the most controversial since its taxonomy has been under ongoing discussion for the past 200 years. According to different authors, it has been divided into practically a single genus [3], through 17 [4,5] to 36 genera [6], with the genus *Maxillaria* Ruiz & Pav. always being the core of the subtribe. Its distribution range is exclusively Neotropical as it covers both Central and South America (with the Caribbean). A large number of taxa and a wide distribution range make Maxillariinae an important Neotropical flora compound and an excellent candidate for further phytochemical studies with potential commercial outcomes.

Studies conducted since the middle of the 20th century revealed a great diversity of labellar epidermis in many groups of orchids. The first attempts to investigate the micromorphological features in *Maxillaria sensu lato* were conducted in 1998 [7], and, since then, several dozen papers have been published (e.g., [8,9,10,11,12,13]). Glabrous labella are not common in *Maxillaria* and tend to occur mainly in species assigned to the *M. cucullata* alliance [14]. The labellar papillae and trichomes of *Maxillaria* show great diversity as they may be conical, obpyriform, villiform, fusiform, or clavate. Labellar papillae may contain protein, lipids, and starch. Many papillae contain pigment or act as osmophores, which may play a role in attracting insects. Some of them may have a protective role in preventing desiccation [14]. Papillae are largely responsible for the production of labellar secretions that may have different chemical compositions. These secretions may contain active compounds of potential medical importance.

**Figure 1:**
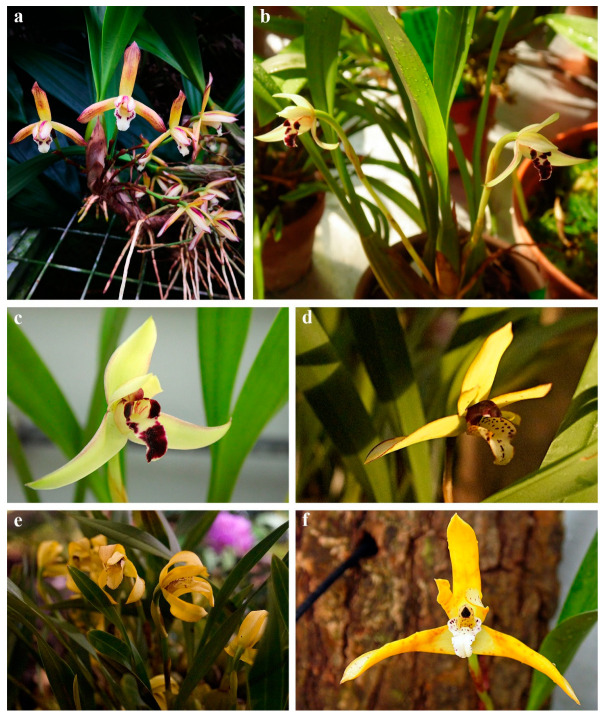
Flowers of *Brasiliorchis* species examined to date: (**a**) *B. gracilis*; (**b,c**) *B. marginata*; (**d**) *B. picta*; (**e**) *B. porphyrostele;* (**f**) *B.* cf. *porphyrostele*. Photo. M. Lipińska.

**Figure 2:**
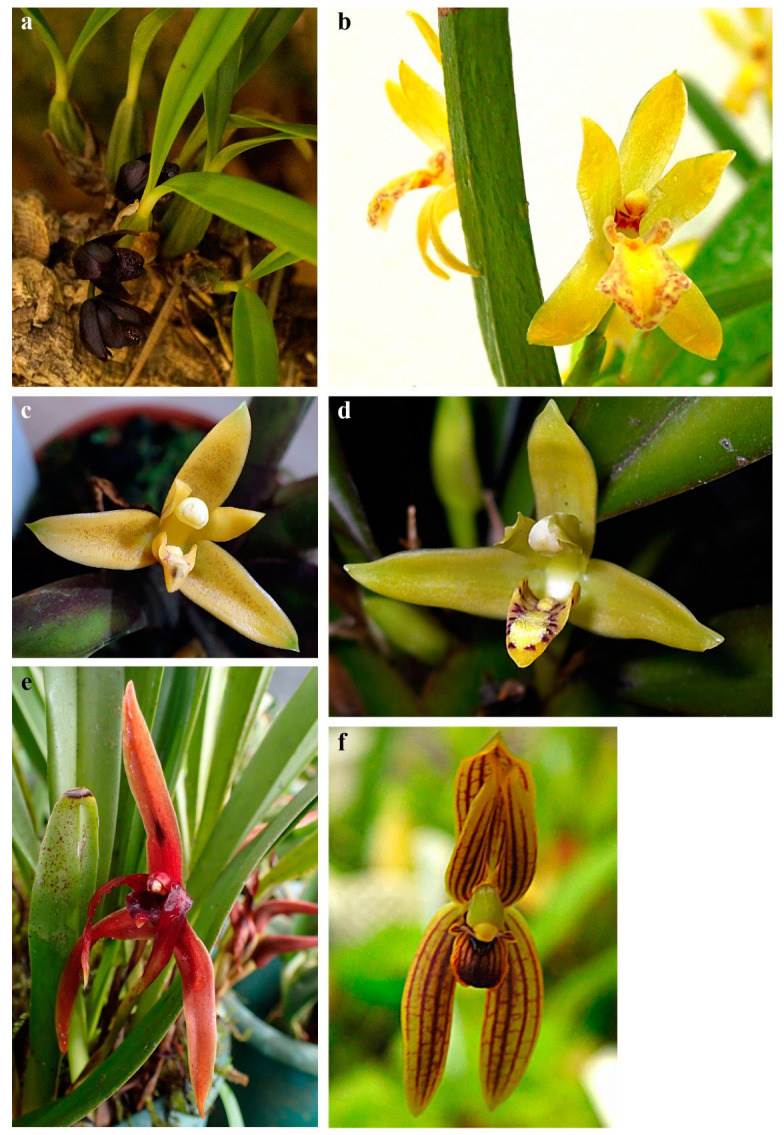
Flowers of *Maxillariinae* species examined to date: (**a**) *B. schunkeana*; (**b**) *Chelyella* sp.; (**c,d**) *Heterotaxis* cf. *discolor*; (**e**) *Maxillaria nigrescens;* (**f**) *Mormolyca ringens*. Photo. M. Lipińska.

**Figure 3:**
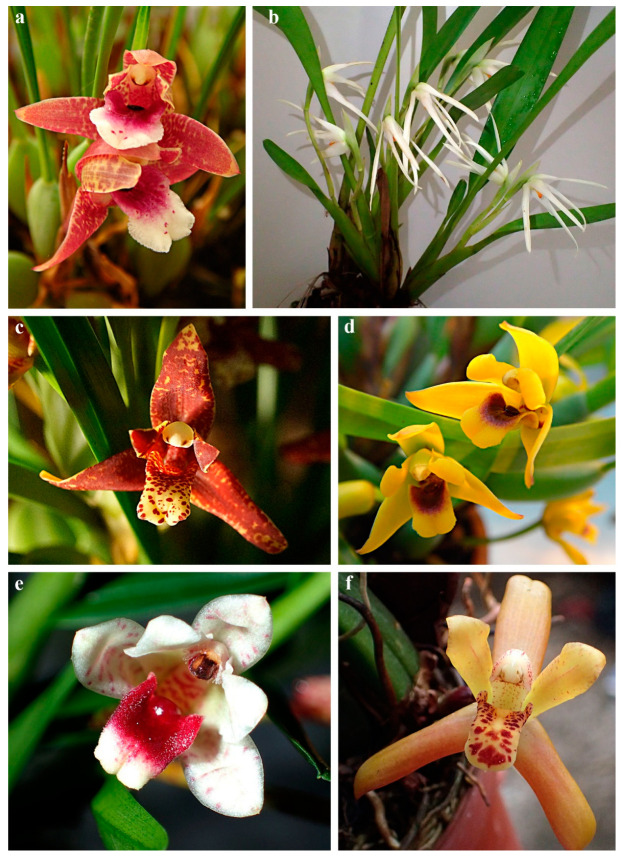
Flowers of Maxillariinae species examined to date: (**a**) *Maxillariella sanguinea*; (**b**) *Maxillaria splendens*; (**c**) *Maxillariella tenuifolia*; (**d**) *M. variabilis*; (**e**) *M. vulcanica*; (**f**) *Xanthoxerampellia rufescens*. Photo. M. Lipińska.

While preparing the presented review we analyzed the literature published between 1991 and 2022 that concerned the chemical composition of extracts and labellar secretions produced by the Maxillariinae subtribe members. To date, only several species have been investigated in this regard: *Brasiliorchis gracilis* (G. Lodd.) R.B. Singer, S. Koehler & Carnevali [15] (Figure 1a), *B. margi*nata (Lindl.) R.B. Singer, S. Koehler & Carnevali [15] (Figure 1b,c), *B. picta* (Hook.) R.B. Singer S. Koehler & Carnevali [15,16,17,18] (Figure 1d), *B. porphyrostele* (Rchb. f.) R.B. Singer, S. Koehler & Carnevali [19] (Figure 1e,f), *B. schunkeana* (Campacci & Kautsky) R.B. Singer, S. Koehler & Carnevali [20] (Figure 2a), *Chelyella densa* (Lindl.) Szlach. & Sitko [21], *Ch. jenischiana* (Rchb. f.) Szlach. & Sitko [15] (Figure 2b), *Heterotaxis discolor* (G. Lodd. ex Lindl.) Ojeda & Carnevali (Lipińska & Haliński, unpbl. data) (Figure 2c,d), *H. superflua* (Rchb. f.) F. Barros [22], *Maxillaria nigrescens* Lindl. [17] (Figure 2e), *M. splendens* Poepp. & Endl. (Lipińska & Haliński, unpbl. data) (Figure 3b), *Maxillariella sanguinea* (Rolfe) M.A. Blanco & Carnevali [23] (Figure 3a), *M. tenuifolia* (Lindl.) M.A. Blanco & Carnevali [16,17,24,25,26] (Figure 3c), *M. variabilis* (Bateman *ex* Lindl.) M.A. Blanco & Carnevali [17,23] (Figure 3d), *M. vulcanica* (F. Lehm. & Kraenzl.) M.A. Blanco & Carnevali [23] (Figure 3e), *Mormolyca ringens* (Lindl.) Schltr. [27] (Figure 2f), *Trigonidium obtusum* Lindl.[15], *Trigonidium* cf. *turbinatum* Rchb. f. [15], and *Xanthoxerampellia rufescens* (Lindl.) Szlach. & Sitko [15, Lipińska & Haliński, unpbl. data] (Figure 3f) (classification *sensu* Szlachetko [6]).

Orchids are widely used in traditional medicine for the treatment of a whole range of different health conditions: skin issues, infectious diseases, digestive problems, respiratory issues, reproduction malfunctions, circulation and heart problems, tumors, pain, and fever. Indeed, throughout the ages, orchid extracts were attributed to some activities such as diuretic, anti-inflammatory, or antimicrobial. For example, Ecuadorian healers (los curanderos) use stem and flower extracts of *Epidendrum secundum* Jacq. to heal nervous disorders and liver diseases [28]. *Stanhopea anfracta* Rolfe is utilized in treating cough and lung diseases thanks to the presence of eucalyptol in its flowers [28]. Some species are used as emetics, aphrodisiacs, vermifuges, bronchodilators, and sex stimulators or to treat scorpion stings and snake bites [29]. Representatives of *Maxillaria sensu lato* are not an exception and are also widely used in traditional medicine for instance for their antispasmodic and anti-inflammatory activities [30].

Within the compounds detected with the use of gas chromatography/mass spectrometry (GC–MS) and liquid chromatography/tandem mass spectrometry (LC–MS/MS) in the tissues of different Maxillariinae representatives (mainly lip secretions), several of them have already been investigated for their medicinal uses (see Table 1). The presented work aimed to summarize published data on semiochemicals that have therapeutic potential and that could be sourced from representatives of Maxillariinae. Additionally, we add information on examples of other sources of these substances (see Appendix A). We hope that this review will lead specialists in the field to design further studies to better understand and exploit orchids, especially Maxillariinae, as sources of biologically active compounds.

## 2. Active Compounds Found in Maxillariinae

### 2.1. Aldehydes

1. Nonanal

(Pelargonaldehyde, 1-nonanal, nonanaldehyde, pelargonic aldehyde, nonylic aldehyde, n-nonanal, 9Ald)

CAS Number: 124-19-6

Occurrence in Maxillariinae: *Brasiliorchis gracilis*, *B. marginata* [15], *B. picta* [18], *Chelyella jenischiana* [15], *Heterotaxis discolor*, *Maxillaria splendens* (Lipińska & Haliński, unpbl. data), *Maxillariella tenuifolia* [25], *Mormolyca ringens* [27], *Trigonidium* cf. *turbinatum* [15], *Xanthoxerampellia rufescens* [15], Lipińska & Haliński, unpbl. data.

Activity: antidiarrheal activity [31]; antimicrobial activity against Gram-positive and Gram-negative bacteria; antifungal activity [32].

### 2.2. Aromatics

1. Benzaldehyde

(Benzoic aldehyde, phenylmethanal, benzenecarboxaldehyde, benzenecarbonal, benzene carbaldehyde, benzaldehyde FFC, benzoic acid aldehyde)

CAS Number: 100-52-7

Occurrence in Maxillariinae: *Brasiliorchis picta* [16,17,18], *Maxillaria nigrescens* [17], *Maxillariella tenuifolia* [16,17], *Xanthoxerampellia rufescens* [15].

Activity: antitumor activity [33]; antibacterial activity against *Staphylococcus aureus*; toxic action against *Drosophila melanogaster* [34].

2. Benzoic acid, 3-methoxy-4-hydroxy

(Vanillic acid, p-Vanillic acid, Acide vanillique, 3-Methoxy-4-hydroxybenzoic acid, Vanillate, VA, VAN)

CAS Number: 121-34-6

Occurrence in Maxillariinae: *Maxillariella sanguinea* [23], *M. tenuifolia* [26], *M. variabilis* [23].

Activity: antioxidative and antimicrobial activity [35,36]; beneficial effect on DSS-induced ulcerative colitis, usefulness in the regulation of chronic intestinal inflammation and effectiveness in the management of immune or inflammatory responses [37]; immunomodulating activities and suppressing effect on hepatic fibrosis in chronic liver injury [38]; neuroprotective agent in the treatment of vascular dementia and cerebrovascular insufficiency states, inflammation, and neurological diseases (e.g., Alzheimer’s disease and Parkinson’s Disease) [39]; significant α-glucosidase-inhibitory activity [26].

3. Benzoic acid, 4-ethoxy-, ethyl ester

(Ethyl 4-ethoxybenzoate; benzoic acid, 4-ethoxy-, ethyl ester; 4-ethoxybenzoic acid ethyl ester; 4-ethoxy ethylbenzoate; benzoic acid, p-ethoxy-, ethyl ester; ethyl p-ethoxybenzoate; ethyl-4-ethoxybenzoate; PEEB; Ethyl para-ethoxybenzoate)

CAS Number: 23676-09-7

Occurrence in Maxillariinae: *Maxillariella sanguinea*, *M. vulcanica* [23].

Activity: antimicrobial and preservative properties [40]; antioxidant and anti-inflammatory properties [41].

4. Butylated hydroxytoluene

(2,6-Di-tert-butyl-4-methylphenol; butylhydroxytoluene; 2,6-di-tert-butyl-p-cresol; 2,6-di-t-butyl-4-methylphenol; BHT; DBPC)

CAS Number: 128-37-0

Occurrence in Maxillariinae: *Brasiliorchis gracilis*, *B. marginata*, *Chelyella jenischiana*, *Trigonidium* cf. *turbinatum* [15]

Activity: antioxidant activity and antiatherogenic effect [42]; induced resistance against *Botryosphaeria dothidea* [43].

5. Cinnamic acid

(3-Phenyl-2-propenoic acid; trans-cinnamic acid; 3-phenylacrylic acid; (E)-cinnamic acid; trans-3-phenylacrylic acid; E-cinnamic acid; phenylacrylic acid; trans-cinnamate; (2E)-3-phenylprop-2-enoic acid)

CAS Number: 621-82-9

Occurrence in Maxillariinae: *Maxillariella sanguinea* [23], *Mormolyca ringens* [27].

Activity: antitumor activity [44]; cytotoxic, cytostatic, antiproliferative, antiangiogenic, and antileukemic; active against solid tumors; inhibit different enzymes, e.g., transglutaminase, aminopeptidase N, and histone deacetylase; cause DNA-damage [45]; inhibitory activity against several Gram-positive and Gram-negative bacteria; antiviral and antifungal properties [46]; antimicrobial [47].

6. Cinnamic acid, 4-hydroxy-3-methoxy

(Ferulic acid; trans-ferulic acid; 4-hydroxy-3-methoxycinnamic acid; trans-4-hydroxy-3-methoxycinnamic acid; 3-(4-hydroxy-3-methoxyphenyl)acrylic acid; (E)-ferulic acid; ferulate; coniferic acid)

CAS Number: 537-98-4

Occurrence in Maxillariinae: *Maxillariella sanguinea* [23].

Activity: antioxidant potential [48]; antioxidant, antimicrobial, anti-inflammatory, anti-thrombosis, and anticancer activities; protection against coronary disease; lowers cholesterol and increases sperm viability ([49] and references therein); potent antitumor agent ([45] and references therein); potent function in muscle cell proliferation, differentiation, and development [50].

7. Indole

(1H- indole)

CAS Number: 120-72-9

Occurrence in Maxillariinae: *Brasiliorchis picta* [17], *Heterotaxis discolor* (Lipińska & Haliński, unpbl. data), *Maxillaria nigrescens*, *Maxillariella tenuifolia*, *M. variabilis* [17].

Activity: antibacterial and anticancer activities [51].

8. p-Anisaldehyde

(4-Methoxybenzaldehyde; anisic aldehyde; anisaldehyde; p-methoxybenzaldehyde; 4-anisaldehyde; benzaldehyde, 4-methoxy-; p-formylanisole)

CAS Number: 123-11-5

Occurrence in Maxillariinae: *Brasiliorchis picta* [17], *Chelyella jenischiana* [15], *Maxillaria nigrescens*, *Maxillariella tenuifolia*, *M. variabilis* [17].

Activity: acaricidal activity against *Dermatophagoides farinae* and *D. pteronyssinus* [52]; repellent effects [53]; antimicrobial [54]; antibacterial and antioxidant activity [55].

### 2.3. Carboxylic Acids

1. Azelaic acid

(Nonanedioic acid; anchoic acid; 1,7-heptanedicarboxylic acid; 1,9-nonanedioic acid; heptanedicarboxylic acid; n-nonanedioic acid)

CAS Number: 123-99-9

Occurrence in Maxillariinae: *Brasiliorchis schunkeana* [20], *Chelyella jenischiana* [15], *Maxillariella sanguinea*, *M. variabilis*, *M. vulcanica* [23].

Activity: bacteriostatic and bactericidal properties against a variety of aerobic and anaerobic microorganisms; effective in the treatment of comedonal acne and inflammatory (papulopustular, nodular, and nodulocystic) acne, as well as various cutaneous hyperpigmentary disorders characterized by hyperactive/abnormal melanocyte function, including melasma and, possibly, lentigo maligna; antiproliferative and cytotoxic effect on the human malignant melanocyte; preliminary findings indicate that it may arrest the progression of cutaneous malignant melanoma [56].

2. Nonanoic acid

(Pelargonic acid; n-nonanoic acid; nonoic acid; nonylic acid; 1-octanecarboxylic acid; pelargon)

CAS Number: 112-05-0

Occurrence in Maxillariinae: *Maxillariella sanguinea*, *M. vulcanica* [23].

Activity: antibiofilm [57]; antifungal activity [57,58].

3. Octanoic acid

(Caprylic acid; n-octanoic acid; octylic acid; n-caprylic acid; octoic acid; n-octylic acid; n-octoic acid; 1-heptanecarboxylic acid; enantic acid; octic acid)

CAS Number: 124-07-2

Occurrence in Maxillariinae: *Maxillariella sanguinea*, *M. vulcanica* [23].

Activity: effective in inactivating infant pathogens such as herpes simplex virus, respiratory syncytial virus, *Haemophilus influenzae*, and Group B streptococci [59]; bactericidal against the major bovine mastitis pathogens *Streptococcus agalactiae*, *S. dysgalactiae*, *S. uberis*, *S. aureus,* and *E. coli* [60]; potential fatty acid chemotherapeutic for glioblastoma [61]; antifungal properties [62].

4. Suberic acid

(Octanedioic acid; 1,8-octanedioic acid; 1,6-hexanedicarboxylic acid; hexamethylenedicarboxylic acid; octane-1,8-dioic acid; 1,6-dicarboxyhexane; cork acid)

CAS Number: 505-48-6

Occurrence in Maxillariinae: *Brasiliorchis schunkeana* [20], *Maxillariella sanguinea*, *M. variabilis*, *M vulcanica* [23].

Activity: anti-photoaging agent [63].

### 2.4. Fatty Acids and Their Esters

1. Oleic acid

(9-Octadecenoic acid; (Z)-9-octadecenoic acid; cis-9-octadecenoic acid; oleate; (Z)-octadec-9-enoic acid; elaidoic acid; cis-oleic acid)

CAS Number: 112-80-1

Occurrence in Maxillariinae: *Maxillariella sanguinea* [23], *M. tenuifolia* [25], *M. vulcanica* [23].

Activity: inhibition of *Streptococcus aureus* primary adhesion [64]; strong antibacterial and antibiofilm activities against *Porphyromonas gingivalis*, a bacterial pathogen involved in chronic periodontitis; inhibits the early stage of biofilm formation by this organism [65]; cytotoxic to bacteria, with a potentially strong effect against Gram-negative bacterium *Klebsiella pneumonia* [66].

2. Heptadecanoic acid

(Margaric acid; n-Heptadecanoic acid; n-heptadecylic acid; heptadecylic acid; n-heptadecoic acid)

CAS Number: 506-12-7

Occurrence in Maxillariinae: *Maxillariella sanguinea* [23].

Activity: a biomarker for coronary heart disease (CHD) risk and type 2 diabetes mellitus (T2D) risk; evidence for theories of alternate endogenous metabolic pathways [67].

3. Hexadecanoic acid

(Palmitic acid; 1-pentadecanecarboxylic acid; pentadecanecarboxylic acid; hexadecanoate; hexaectylic acid; 1-hexyldecanoic acid; hexadecoic acid)

CAS Number: 57-10-3

Occurrence in Maxillariinae: *Maxillariella sanguinea*, *M. variabilis*, *M. vulcanica* [23].

Activity: anti-inflammatory activity [68]; anticancer cytotoxic potential [69]; potential antioxidant and anticancer activity [70].

4. Tetradecanoic acid

(Myristic acid; n-tetradecanoic acid; n-tetradecan-1-oic acid)

CAS Number: 544-63-8

Occurrence in Maxillariinae: *Maxillariella sanguinea*, *M. variabilis*, *M. vulcanica* [23].

Activity: larvicidal and repellent activity against *Aedes aegypti* and *Culex quinquefasciatus* [71].

5. Octadecanoic acid, methyl ester

(Methyl stearate; methyl octadecanoate; stearic acid methyl ester; methyl n-octadecanoate)

CAS Number: 112-61-8

Occurrence in Maxillariinae: *Brasiliorchis schunkeana* [20], *Maxillariella variabilis*, *M. vulcanica* [23].

Activity: antiviral activity [72].

### 2.5. Hydrocarbons

1. 4,8,8-Trimethyl-2-methylene-4-vinylbicyclo[5.2.0]nonane

(2-methylene-4,8,8-trimethyl-4-vinyl-bicyclo[5.2.0]nonane; Bicyclo[5.2.0]nonane, 2-methylene-4,8,8-trimethyl-4-vinyl-)

CAS Number: lack; PubChem CID: 564746

Occurrence in Maxillariinae: *Maxillariella tenuifolia* [25].

Activity: heat-clearing and detoxifying effects; potential anti-influenza activity [73].

2. Heptacosane

(n-Heptacosane; 27Hy)

CAS Number: 593-49-7

Occurrence in Maxillariinae: *Maxillariella sanguinea* [23].

Activity: modulator of P-gp in a model of AML multidrug resistant HL-60R [74].

### 2.6. Ketones

1. 2-Pentadecanone

(Pentadecan-2-one; methyl tridecyl ketone)

CAS Number: 2345-28-0

Occurrence in Maxillariinae: *Maxillariella tenuifolia* [25].

Activity: antibacterial activity against *Staphylococcus aureus*; wound closure; collagen deposition; fibroblast proliferation effects; potency to be used as an active ingredient in the formulation of a diabetic wound-healing cream [75]; positive effect on the skin wound healing process; inhibition of ethanol-induced mucosal ulceration based on antioxidant activity; diminishing inflammation; upregulation of Hsp70 and downregulation of Bax protein in skin and stomach tissue; support collagen synthesis in skin tissue and mucus production in the stomach [76].

2. 2-Undecanone

(Undecan-2-one; methyl nonyl ketone; 2-hendecanone; undecanone; rue ketone; ketone, methyl nonyl)

CAS Number: 112-12-9

Occurrence in Maxillariinae: *Maxillaria tenuifolia* [24,25].

Activity: cytotoxicity against human carcinoma cells [77]; cytotoxicity against *Leishmania* protozoans [78]; causes plasma membrane malformations and intensive vacuolation of cytoplasm in *Aspergillus flavus* [79]; activity against *Caenorhabditis elegans, Drosophila melanogaster*, and *Rhizoctonia solani* [80]; can significantly reduce B[a]P-induced DNA damage and inflammation to prevent lung tumorigenesis by activating the Nrf2/HO-1/NQO-1 signaling pathway; may exert beneficial effects against cigarette smoke-induced lung inflammation and oxidative DNA damage in the human body and, thus, could be an effective candidate agent for the chemoprevention of lung cancer [81]; anti-inflammatory properties; can induce kidney inflammation; by inducing mitophagy, may play a protective role against renal inflammation [82]; insect repellent; antibiofilm and anti-hyphal potential [83].

### 2.7. Monoterpenes

1. 4-Terpineol

(4-Carvomenthenol; terpene-4-ol; terpinen-4-ol; 1-terpinen-4-ol; terpinenol-4; p-menth-1-en-4-ol; 1-p-menthen-4-ol)

CAS Number: 562-74-3

Occurrence in Maxillariinae: *Heterotaxis discolor*, *Xanthoxerampellia rufescens* (Lipińska & Haliński, unpbl. data).

Activity: anticancer effects in Hep-G2 [84]; activity against various microorganisms, such as *Streptococcus aureus*, *Pseudomonas aeruginosa,* and coagulase-negative staphylococci (CoNS); antifungal effect against fungi such as *Candida* spp., *Saccharomyces cerevisiae*, *Trichophyton rubru*, and *Penicillium* spp.; miticidal effect against *Demodex* mites, which play a role in blepharitis, unexplained keratitis, superficial corneal vascularization, marginal infiltration, phlyctenule-like lesions, nodular scarring, and rosacea; anti-inflammatory properties by suppressing superoxide production and proinflammatory cytokines ([85] and references therein).

2. cis-β-Ocimene

((Z)-3,7-Dimethyl-1,3,6-octatriene; (3Z)-3,7-dimethylocta-1,3,6-triene; (Z)-beta-ocimene; beta-cis-ocimene; cis-3,7-dimethyl-1,3,6-octatriene)

CAS Number: 3338-55-4

Occurrence in Maxillariinae: *Brasiliorchis gracilis* [15], *B. picta* [16,17,18], *Maxillaria nigrescens* [17], *Maxillariella tenuifolia* [16,17], *M. variabilis* [16].

Activity: potential as an antifungal agent against a wide spectrum of fungal species frequently implicated in human mycoses, particularly candidiasis, cryptococcosis, and dermatophytosis [86].

3. Limonene

(1-Methyl-4-(1-methylethenyl)-cyclohexene; p-mentha-1,8-diene; 1,8-p-menthadiene; cyclohexene, 1-methyl-4-(1-methylethenyl)-; dipentene)

CAS Number: 138-86-3

Occurrence in Maxillariinae: *Brasiliorchis gracilis*, *B. marginata* [15], *B. picta* [16,17,18], *Heterotaxis discolor* (Lipińska & Haliński, unpbl. data), *Maxillaria nigrescens* [17], *M. splendens* (Lipińska & Haliński, unpbl. data), *Maxillariella sanguinea* [23], *M. tenuifolia* [16,17,24,25], *M. variabilis* [17], *Trigonidium* cf. *turbinatum* [17], *Xanthoxerampellia rufescens* (Lipińska & Haliński, unpbl. data).

Activity: dissolving gallstones [87]; antimicrobial properties against various bacteria, e.g., *Escherichia coli* and *Bacillus cereus*, and yeast *Cryptococcus neoformans* [88]; chemotherapeutic agent for breast cancer [89]; preventive and ameliorating effects on dyslipidemia and hyperglycemia [90]; antibiofilm potential against *Streptococcus* spp. [91]; gastroprotection through local mucosal defense mechanisms, such as increased mucus production, modulation of the oxidative stress and inflammatory response [92]; potent anticancer agent against human bladder cancer [93]; anti-inflammatory and antioxidant properties [94].

4. Eucalyptol

(1,3,3-Trimethyl-2-oxabicyclo[2.2.2.]octane; cineole; 1,8-cineole; 1,8-cineol)

CAS Number: 470-82-6

Ocurrence in Maxillariinae: *Brasiliorchis picta* [16,17,18], *Maxillaria nigrescens* [17], *Maxillariella tenuifolia* [16,17,24,25], *M. variabilis* [17].

Activity: dehumidification, insecticide, and analgesia activity [95]; attenuation of cerulein-induced acute pancreatitis via an anti-inflammatory mechanism and by combating oxidative stress [96]; anti-inflammatory and antioxidant activity mainly via the regulation of NF-κB and Nrf2, an important role in the treatment of cardiovascular illness, cancers, digestive disorders, Alzheimer’s disease (AD); respiratory ailments such as bronchitis, asthma, and chronic obstructive pulmonary disease (COPD); bacilli ([97] and references therein).

5. γ-terpinene

(gamma-Terpinene; 1,4-p-menthadiene; 1-isopropyl-4-methyl-1,4-cyclohexadiene; 1-isopropyl-4-methylcyclohexa-1,4-diene;1-methyl-4-(1-methylethyl)-1,4 cyclohexadiene; 1-methyl-4-(propan-2-yl)cyclohexa-1,4-diene; 4-Isopropyl-1-methyl-1,4-cyclohexadiene)

CAS Number: 99-85-4

Occurrence in Maxillariinae: *Brasiliorchis picta* [17,18], *Heterotaxis discolor* (Lipińska & Haliński, unpbl. data), *Maxillaria nigrescens* [17], *Maxillariella tenuifolia* [17,24], *M. variabilis* [17], *Xanthoxerampellia rufescens* (Lipińska & Haliński, unpbl. data).

Activity: anti-inflammatory properties [98]; antibacterial, antifungal, and anticancer properties [99].

6. Linalool

(2,6-Dimethyl-2,7-octadien-6-ol; 3,7-dimethylocta-1,6-dien-3-ol; linalol; 3,7-dimethyl-1,6-octadien-3-ol; allo-ocimenol; beta-linalool; 1,6-octadien-3-ol, 3,7-dimethyl-)

CAS Number: 78-70-6

Occurrence in Maxillariinae: *Brasiliorchis gracilis*, *B. maginata* [15], *B. picta* [15,16,17,18], *Maxillaria nigrescens* [17], *Maxillariella tenuifolia* [16,17], *M. variabilis* [17].

Activity: analgesic activity [100]; antibacterial, antifungal, antioxidant, anti-inflammatory, and anticancer activities [101].

7. p-Cymene

(1-Methyl-4-(1-methylethyl)-benzene; 1-isopropyl-4-methylbenzene; 4-isopropyltoluene; p-isopropyltoluene; para-cymene; p-cymol)

CAS Number: 99-87-6

Occurrence in Maxillariinae: *Brasiliorchis gracilis* [15], *B. picta* [16,17], *Chelyella jenischiana* [15], *Maxillaria nigrescens* [17], *Maxillariella tenuifolia* [16,17], *M. variabilis* [17], *Trigonidium* cf. *turbinatum* [15].

Activity: analgesic-like property ([102] and references therein); antioxidant, anti-inflammatory, antinociceptive, anxiolytic, anticancer, and antimicrobial effects ([103] and references therein); antidiabetic, anti-enzymatic, antiparasitic, immunomodulatory, vasorelaxant, and neuroprotective agent ([104] and references therein).

8. α-Pinene

(Alpha-pinene; 2-pinene; acintene a; .alpha.-pinene; 2,6,6-trimethylbicyclo[3.1.1]hept-2-ene; (+/−)-alpha-pinene; bicyclo[3.1.1]hept-2-ene, 2,6,6-trimethyl-)

CAS Number: 80-56-8

Occurrence in Maxillariinae: *Brasiliorchis marginata* [15], *B. picta* [15,16,17,18], *Heterotaxis discolor* (Lipińska & Haliński, unpbl. data), *Maxillaria nigrescens* [17], *Maxillariella tenuifolia* [16,17,24], *M. variabilis* [17], *Xanthoxerampellia rufescens* (Lipińska & Haliński, unpbl. data).

Activity: antimicrobial and antibiofilm formation; activity against *Candida albicans*, *Cryptococcus neoformans*, *Rhizopus oryzae*, and *Staphylococcus aureus* MRSA [105]; antimicrobial, anticancer, anti-inflammatory, and antiallergic properties; cytogenetic, gastroprotective, anxiolytic, cytoprotective, anticonvulsant, and neuroprotective effects, as well as effects against H_2_O_2_-stimulated oxidative stress, pancreatitis, stress-stimulated hyperthermia, and pulpal pain [106].

9. α-Terpineol

((.+/−.)-.alpha.-Terpineol; .alpha.,.alpha.,4-trimethyl-3-cyclohexene-1-methanol; 1-p-menthen-8-ol; 2-(4-methyl-3-cyclohexen-1-yl)-2-propanol; 2-(4-methylcyclohex-3-enyl)-propan-2-ol; 3-cyclohexene-1-methanol, .alpha.,.alpha.4-trimethyl-; 4-(2-hydroxy-2-propyl)-1-methylcyclohexene)

CAS Number: 98-55-5

Occurrence in Maxillariinae: *Brasiliorchis picta* [16,17,18], *Heterotaxis discolor* (Lipińska & Haliński, unpbl. data), *Maxillaria nigrescens* [17], *Maxillariella tenuifolia* [16,17], *M. variabilis* [17], *Xanthoxerampellia rufescens* (Lipińska & Haliński, unpbl. data).

Activity: cardiovascular and antihypertensive effects; antioxidant, anticancer, antinociceptive, antiulcer, anticonvulsant, sedative, anti-bronchitis, skin penetration enhancing, and insecticidal activities [107,108].

10. β-Pinene

(6,6-Dimethyl-2-methylenebicyclo[3.1.1]heptane)

CAS Number: 127-91-3

Occurrence in Maxillariinae: *Brasiliorchis picta* [16,17,18], *Maxillaria nigrescens* [17], *Maxillariella tenuifolia* [16,17,24,25], *M. variabilis* [17], *Trigonidium* cf. *turbinatum* [15].

Activity: antimicrobial and antibiofilm formation activity against *Candida albicans*, *Cryptococcus neoformans*, *Rhizopus oryzae*, and *Staphylococcus aureus* MRSA [105]; antimicrobial, anticancer, anti-inflammatory, and antiallergic properties; cytogenetic, gastroprotective, anxiolytic, cytoprotective, anticonvulsant, and neuroprotective effects, as well as effects against H_2_O_2_-stimulated oxidative stress, pancreatitis, stress-stimulated hyperthermia, and pulpal pain [106].

### 2.8. Sesquiterpenes

1. ar-Curcumene

(1-Methyl-4-(6-methylhept-5-en-2-yl)-benzene)

CAS Number: 4176-17-4

Occurrence in Maxillariinae: *Brasiliorchis marginata*, *Chelyella jenischiana* [15], *Mormolyca ringens* [27], *Trigonidium* cf. *turbinatum*, *Xanthoxerampellia rufescens* [15].

Activity: potential protective effect on LPS-stimulated BEAS-2B cells regarding IL-8 and RANTES secretion and might serve as drugs against inflammatory airway diseases [109].

2. Aromadendrene

(1,1,7-Trimethyl-4-methylenedecahydro-1H-cyclopropa[e]azulene; alloaromadendrene)

CAS Number: 109119-91-7

Occurrence in Maxillariinae: *Brasiliorchis gracilis*, *B. marginata* [15], *B. picta* [16,17], *Chelyella jenischiana* [15], *Heterotaxis discolor* (Lipińska & Haliński, unpbl. data), *Maxillaria nigrescens* [17], *Maxillariella tenuifolia* [16,17,24,25], *M. variabilis* [17], *Mormolyca ringens* [27], *Trigonidium* cf. *turbinatum* [15], *Xanthoxerampellia rufescens* [15] (Lipińska & Haliński, unpbl. data).

Activity: antimicrobial activity [95].

3. Calarene

CAS Number: 13466-78-9

Occurrence in Maxillariinae: *Maxillariella tenuifolia* [24].

Activity: larvicidal activity against *Anopheles stephensi*, *Aedes aegypti*, and *Culex quinquefasciatus* (against malaria, dengue, yellow fever, and filariasis mosquitos) [110].

4. Caryophylladienol II

(Caryophylla-2(12);6(13)-dien-5beta-ol)

CAS Number: 19431-79-9

Occurrence in Maxillariinae: *Maxillariella tenuifolia* [25].

Activity: probable antimicrobial activity against the Gram-positive bacteria *Staphylococcus aureus* and *Bacillus cereus* [111].

5. Caryophyllene oxide

CAS Number: 1139-30-6

Occurrence in Maxillariinae: *Brasiliorchis picta* [16,17], *Maxillaria nigrescens* [17], *Maxillariella tenuifolia* [16,17,25], *M. variabilis* [17].

Activity: analgesic and anti-inflammatory activity [112]; anticancer, enhancing the efficacy of some chemotherapeutics [113]; anticholinesterase and antioxidant capacities [114]; treatment of onychomycosis [115]; induction of apoptotic cell death in prostate cancer cells [116].

6. Caryophyllene

(Decahydro-2,2,4,8-tetramethyl-4,8-methanoazulen-9-ol)

CAS Number: 4586-22-5

Occurrence in Maxillariinae: *Brasiliorchis gracilis*, *B. marginata* [15], *B. picta* [16,17,18], *Chelyella jenischiana* [15], *Maxillaria nigrescens* [17], *M. splendens* (Lipińska & Haliński, unpbl. data), *Maxillariella tenuifolia* [16,17,24,25], *M. variabilis* [17].

Activity: significantly increasing the anticancer activity of α-humulene and isocaryophyllene on MCF-7 cells; anticarcinogenic activity [117]; selective antibacterial activity against *S. aureus*, antifungal activity, strong antioxidant effects, and selective antiproliferative effects against colorectal cancer cells [118]; anti-inflammatory, anticarcinogenic, antimicrobial, antioxidative, and analgesic activities; strong cytotoxicity against cancer cell lines (HCT-116, HT-29, colon cancer; PANC-1, pancreatic cancer) [113].

7. epi-Cubebol

CAS Number: 38230-60-3

Occurrence in Maxillariinae: *Maxillariella tenuifolia* [25].

Activity: potential as a source for natural larvicides (activity against Aedes aegypti and A. albopictus) [119].

8. α-Copaene

(8-Isopropyl-1,3-dimethyl-tricyclo[4.4.0.0(2,7)]dec-3-ene)

CAS Number: 3856-25-5

Occurrence in Maxillariinae: *Brasiliorchis gracilis*, *B. marginata*, *B. picta* [16,17], *Chelyella jenischiana* [15], *Maxillaria nigrescens* [17], *Maxillariella sanguinea* [23], *M. tenuifolia* [16,17,24,25], *M. variabilis* [17], *Mormolyca ringens* [27], *Trigonidium* cf. *turbinatum*, *Xanthoxerampellia rufescens* [15].

Activity: nongenotoxic/mutagenic feature, weak antioxidant, and cytotoxic activity; potential in application in anticancer therapy; anticarcinogenic, antioxidant, hepatoprotective, and anti-inflammatory potential; antigenotoxic and antioxidant activity ([120] and references therein).

9. α-Humulene

(α-Caryophyllene, trans,trans,trans-2,6,6,9-tetramethyl-1,4,8-cycloundecatriene)

CAS Number: 6753-98-6

Occurrence in Maxillariinae: *Brasiliorchis gracilis* [15], *B. picta* [16,17], *Maxillaria nigrescens* [17], *Maxillariella tenuifolia* [16,17,24], *M. variabilis* [17].

Activity: inhibition of tumor cell growth [121]; anti-inflammatory properties; potential in the treatment of asthma and related inflammatory and allergic diseases ([122] and references therein); inhibition of the growth of *Bacteroides fragilis* cells and biofilms [123]; antitumor and cytotoxic activity against cancer cells; effective against a wide range of microorganisms, in addition to acting as anti-inflammatories by activating or inactivating several factors involved in the inflammatory process; gastroprotective, cicatrizing, analgesic, and antioxidant potentials [124].

10. β-Elemene

(2,4-Diisopropenyl-1-methyl-1-vinylcyclohexane)

CAS Number: 515-13-9

Occurrence in Maxillariinae: *Brasiliorchis gracilis*, *B. marginata* [15], *B. picta* [17], *Chelyella jenischiana* [15], *Maxillaria nigrescens* [17], *Maxillariela tenuifolia* [17,24,25], *M. variabilis* [17], *Trigonidium* cf. *turbinatum*, *Xanthoxerampellia rufescens* [15].

Activity: excellent antitumor activity against several cancer cell lines (PC-3, A549, U87MG, U251, and HCT116); inhibition of tumor cell migration; relatively minor adverse effects [125,126].

11. β-Gurjunene

CAS Number: 17334-55-3

Occurrence in Maxillariinae: Brasiliorchis gracilis, B. marginata, Chelyella jenischiana [15].

Activity: antibacterial activity [127].

12. β-Myrcene

(7-methyl-3-methylene-1,6-octadiene)

CAS Number: 123-35-3

Occurrence in Maxillariinae: *Brasiliorchis picta* [16,17], *Heterotaxis discolor* (Lipińska & Haliński, unpbl. data), *Maxillaria nigrescens* [17], *Maxillariella tenuifolia* [16,17], *M. variabilis* [17], *Xanthoxerampellia rufescens* (Lipińska & Haliński, unpbl. data).

Activity: antioxidant activity [128].

13. δ-Cadinene

((1S,8aR)-4,7-Dimethyl-1-(propan-2-yl)-1,2,3,5,6,8a-hexahydronaphthalene)

CAS Number: 483-76-1

Occurrence in Maxillariinae: *Brasiliorchis gracilis*, *B. marginata* [15], *B. picta* [15,17], *Chelyella jenischiana* [15], *Maxillaria nigrescens* [17], *Maxillariella tenuifolia* [17,24,25], *M. variabilis* [17], *Trigonidium* cf. *turbinatum*, *Xanthoxerampellia rufescens* [15].

Activity: antimicrobial activity against *Streptococcus pneumoniae* [129].

14. δ-Elemene

(3-Isopropenyl-1-isopropyl-4-methyl-4-vinyl-1-cyclohexene)

CAS Number: 20307-84-0

Occurrence in Maxillariinae: *Brasiliorchis gracilis*, *B. marginata*, *Chelyella jenischiana*, *Trigonidium* cf. *turbinatum* [15].

Activity: inducer of cell apoptosis in human lung carcinoma cells by inhibiting the NF-κB pathway [130].

15. Isocaryophyllene

((Z,1S,9R)-4,11,11-Trimethyl-8-methylenebicyclo[7.2.0]undec-4-ene)

CAS Number: 118-65-0

Occurrence in Maxillariinae: *Brasiliorchis picta* [16,17], *Maxillaria nigrescens* [17], *Maxillariella tenuifolia* [16,17,24], *M. variabilis* [17].

Activity: antimicrobial [131]; anticancer activity [117].

### 2.9. Phenanthrene Derivatives

1. Erianthridin

(9,10-dihydro-2,7-dihydroxy-3,4-dimethoxyphenanthrene)

CAS Number: 101508-48-9

Occurrence in Maxillariinae: *Chelyella densa* [21,132].

Activity: spasmolytic activity [29,132]; antinociceptive [133] and anti-inflammatory effect [133,134]; vasorelaxant activity [135]; antitumor effect on lung cancer cell apoptosis [136,137].

2. Fimbriol A

(3,4,9-Trimethoxyphenanthrene-2,5-diol; 2,5-dihydroxy-3,4,9-trimethoxyphenanthrene)

CAS Number: 152841-83-3

Occurrence in Maxillariinae: *Chelyella densa* [21].

Activity: spasmolytic activity [29,132]; antinociceptive [133]; anti-inflammatory effect [133,134]; vasorelaxant activity [135]; significant anti-aggregation activity [138].

3. Flavanthridin

(3,7-dihydroxy-2,4-dimethoxy-9,10-dihydrophenanthrene)

CAS number: 4773-96-0

Occurrence in Maxillariinae: *Maxillariella tenuifolia* [26].

Activity: significant α-glucosidase-inhibitory activity [26].

4. Gymnopusin

(2,7-Dihydro-3,4,9-trimethoxy-phenanthrene; 3,4,9-trimethoxy-2,7-phenanthrenediol; 2,7-phenanthrenediol, 3,4,9-trimethoxy-; 3,4,9-trimethoxyphenanthrene-2,7-diol)

CAS Number: 113476-61-2

Occurrence in Maxillariinae: *Chelyella densa* [21].

Activity: spasmolytic activity [29,139]; vasorelaxant activity [135].

5. Nudol

(2,7-Phenanthrenediol, 3,4-dimethoxy-; 3,4-dimethoxyphenanthrene-2,7-diol; 2,7-dihydroxy-3,4-dimethoxyphenanthrene)

CAS Number: 86630-46-8

Occurrence in Maxillariinae: *Chelyella densa* [21,132].

Activity: spasmolytic activity [29]; potential against osteosarcoma [140].

6. 2,5-dihydroxy-3,4-dimethoxyphenanthrene

CAS Number: not available

Occurrence in Maxillariinae: *Chelyella densa* [21,132].

Activity: spasmolytic activity [132].

### 2.10. Phenol Derivatives

1. 2-Methoxy-4-vinylphenol

(2M4VP; 4-vinylguaiacol; p-vinylguaiacol)

CAS Number: 7786-61-0

Occurrence in Maxillariinae: *Maxillariella sanguinea*, *M. variabilis* [23].

Activity: potent anti-inflammatory effects by inhibiting LPS-induced NO, PGE2, iNOS, and COX-2 in RAW264.7 cells [141]; anticancer effects on pancreatic cancer cell lines (Panc-1 and SNU-213) by reducing their viability by inhibiting the expression of the cell nuclear antigen (PCNA) protein and suppressing the migratory activity of both cell lines [142].

2. Luteolin-6-C-glucoside

(isoorientin; homoorientin)

CAS Number: 4261-42-1

Occurrence in Maxillariinae: *Heterotaxis superflua* [22].

Activity: myolytic activity on smooth muscle-containing preparations from the rat and the guinea pig [143]; certain antimicrobial activity against *Staphylococcus aureus*, *Bacillus subtilis*, and *Pseudomonas aeruginosa* [144]; anticancer and antioxidant activity [145].

3. Gigantol

(5-[2-(3-hydroxy-5-methoxyphenyl)ethyl]-2-methoxyphenol)

CAS Number: 67884-30-4

Occurrence in Maxillariinae: *Chelyella densa* [21].

Activity: inhibition of the LPS-induced iNOS and COX-2 expression via NF-κB inactivation in RAW 264.7 macrophages cells [146]; spasmolytic activity [139]; protective effects against high glucose-evoked nephrotoxicity [147]; attenuates the metastasis of human bladder cancer cells, possibly through Wnt/EMT signaling [148].

### 2.11. Sterols

1. Campesterol

((24R)-24-Methylcholest-5-en-3b-ol)

CAS Number: 474-62-4

Occurrence in Maxillariinae: *Maxillareilla sanguinea* [23].

Activity: cholesterol-lowering and anticarcinogenic effects; antiangiogenic action of campesterol via inhibition of endothelial cell proliferation and capillary differentiation; exhibits chemopreventive effects against many cancers, including prostate, lung, and breast cancers ([149] and references therein).

2. Stigmasterol

(Stigmasta-5,22-dien-3b-ol)

CAS Number: 83-48-7

Occurrence in Maxillariinae: *Maxillareilla sanguinea* [23].

Activity: thyroid-inhibitory and insulin-stimulatory nature; antidiabetic and antiperoxidative properties [150]; potential anti-inflammatory and anticatabolic properties [151].

### 2.12. Others

1. 2,5-di-tert-Butyl-1,4-benzoquinone

CAS Number: 2460-77-7

Occurrence in Maxillariinae: *Brasiliorchis schunkeana* [20], *Maxillariella vulcanica* [23].

Activity: potent antibacterial agent which inhibits the RNA polymerase enzyme [152,153]; potent antiplasmodial activity [154].

2. 7,9-Di-tert-butyl-1-oxaspiro(4,5)deca-6,9-diene-2,8-dione

CAS Number: 82304-66-3

Occurrence in Maxillariinae: *Brasiliorchis schunkeana* [20], *Maxillariella sanguinea* [23], *M. tenuifolia* [25], *M. vulcanica* [23].

Activity: steroidal anti-mineralocorticoid activity and anti-androgen, weak progesterone properties, with some indirect estrogen and glucocorticoid effects [155]; used primarily as a diuretic and antihypertensive, to treat heart failure and ascites in patients with liver disease, lowering hypertension, hypokalemia, secondary hyperaldosteronism (such as occurs with hepatic cirrhosis), and Conn’s syndrome (primary hyperaldosteronism); frequently used to treat a variety of skin conditions including hirsutism, androgenic alopecia, acne, and seborrhea in females and male pattern baldness [156]; antioxidant activity; acetylcholinesterase inhibitory potential [157].

3. Geranylacetone

((E)-6,10-Dimethyl-5,9-undecadien-2-one)

CAS Number: 3796-70-1

Occurrence in Maxillariinae: *Brasiliorchis gracilis*, *B. marginata*, *Chelyella jenischiana* [15], *Maxillariella tenuifolia* [17,24], *Xanthoxerampellia rufescens* [15].

Activity: antitrypanosomal activity; strong repellent against ticks; acts as a deterrent against the Asian larch bark beetle ([158] and references therein); trypanostatic activity [159].

4. Mangiferin

(1,3,6,7-tetrahydroxy-2-[(2S,3R,4R,5S,6R)3,4,5-trihydroxy-6(hydroxymethyl)oxan-2-yl]xanthen-9-one)

CAS number: 4773-96-0

Occurrence in Maxillariinae: *Maxillariella tenuifolia* [26].

Activity: antidiabetic and anti-inflammatory abilities; effective inhibitor of NF-κB signaling pathway; probable anticancer effects [160]; antibacterial, antitumor, antiviral, and immunomodulatory activities ([26] and references therein).

## 3. Conclusions

In the presented paper, on the basis of a literature review, we reported the presence of 62 biologically active compounds produced by Maxillariinae representatives. We divided them into 12 categories: aldehydes (one), aromatics (eight), carboxylic acids (four), fatty acids and their esters (five), hydrocarbons (two), ketones (two), monoterpenes (10), sesquiterpenes (15), phenanthrene derivatives (six), phenol derivatives (three), sterols (two), and others (four). Even though the number of species examined to date is extremely scarce (19 species investigated of ca. 600 belonging to the subtribe), it can already be noted that Maxillariinae representatives are a promising source of biologically active compounds with medical potential, and further investigations are urgently needed.

## Figures and Tables

**Table 1:** List of the active compounds detected in Maxillariinae representatives.

Compound	Maxillariinae Species
Nonanal	*Brasiliorchis gracilis*, *B. marginata*, *B. picta*, *Chelyella jenischiana*, *Heterotaxis discolor*, *Maxillaria splendens*, *Maxillariella tenuifolia*, *Mormolyca ringens*, *Trigonidium* cf. *turbinatum*, *Xanthoxerampellia rufescens*
Benzaldehyde	*Brasiliorchis picta*, *Maxillaria nigrescens*, *Maxillariella tenuifolia*, *Xanthoxerampellia rufescens*
Benzoic acid, 3-methoxy-4-hydroxy	*Maxillariella sanguinea*, *M. tenuifolia*, *M. variabilis*
Benzoic acid, 4-ethoxy-, ethyl ester	*Maxillariella sanguinea*, *M. vulcanica*
Butylated hydroxytoluene	*Brasiliorchis gracilis*, *B. marginata*, *Chelyella jenischiana*, *Trigonidium* cf. *turbinatum*
Cinnamic acid	*Maxillariella sanguinea*, *Mormolyca ringens*
Cinnamic acid, 4-hydroxy-3-methoxy	*Maxillariella sanguinea*
Indole	*Brasiliorchis picta*, *Heterotaxis discolor, Maxillaria nigrescens*, *Maxillariella tenuifolia*, *M. variabilis*
p-Anisaldehyde	*Brasiliorchis picta*, *Chelyella jenischiana*, *Maxillaria nigrescens*, *Maxillariella tenuifolia*, *M. variabilis*
Azelaic acid	*Brasiliorchis schunkeana*, *Chelyella jenischiana*, *Maxillariella sanguinea*, *M. variabilis*, *M. vulcanica*
Nonanoic acid	*Maxillariella sanguinea*, *M. vulcanica*
Octanoic acid	*Maxillariella sanguinea*, *M. vulcanica*
Suberic acid	*Brasiliorchis schunkeana*, *Maxillariella sanguinea*, *M. variabilis*, *M vulcanica*
Oleic acid	*Maxillariella sanguinea*, *M. tenuifolia*, *M. vulcanica*
Heptadecanoic acid	*Maxillariella sanguinea*
Hexadecanoic acid	*Maxillariella sanguinea*, *M. variabilis*, *M. vulcanica*
Tetradecanoic acid	*Maxillariella sanguinea*, *M. variabilis*, *M. vulcanica*
Octadecanoic acid, methyl ester	*Brasiliorchis schunkeana*, *Maxillariella variabilis*, *M. vulcanica*
4,8,8-Trimethyl-2 methylene-4-vinylbicyclo[5.2.0] nonane	*Maxillariella tenuifolia*
Heptacosane	*Maxillariella sanguinea*
2-Pentadecanone	*Maxillariella tenuifolia*
2-Undecanone	*Maxillaria tenuifolia*
4-Terpineol	*Heterotaxis discolor*, *Xanthoxerampellia rufescens*
cis-β-Ocimene	*Brasiliorchis gracilis*, *B. picta*, *Maxillaria nigrescens*, *Maxillariella tenuifolia*, *M. variabilis*
Limonene	*Brasiliorchis gracilis*, *B. marginata*, *B. picta*, *Heterotaxis discolor*, *Maxillaria nigrescens*, *M. splendens*, *Maxillariella sanguinea*, *M. tenuifolia*, *M. variabilis*, *Trigonidium* cf. *turbinatum*, *Xanthoxerampellia rufescens*
Eucalyptol	*Brasiliorchis picta*, *Maxillaria nigrescens*, *Maxillariella tenuifolia*, *M. variabilis*
γ-terpinene	*Brasiliorchis picta*, *Heterotaxis discolor*, *Maxillaria nigrescens*, *Maxillariella tenuifolia*, *M. variabilis*, *Xanthoxerampellia rufescens*
Linalool	*Brasiliorchis gracilis*, *B. maginata*, *B. picta*, *Maxillaria nigrescens*, *Maxillariella tenuifolia*, *M. variabilis*
p-Cymene	*Brasiliorchis gracilis*, *B. picta*, *Chelyella jenischiana*, *Maxillaria nigrescens*, *Maxillariella tenuifolia*, *M. variabilis*, *Trigonidium* cf. *turbinatum*
α-Pinene	*Brasiliorchis marginata*, *B. picta*, *Heterotaxis discolor*, *Maxillaria nigrescens*, *Maxillariella tenuifolia*, *M. variabilis*, *Xanthoxerampellia rufescens*
α-Terpineol	*Brasiliorchis picta*, *Heterotaxis discolor*, *Maxillaria nigrescens*, *Maxillariella tenuifolia*, *M. variabilis*, *Xanthoxerampellia rufescens*
β-Pinene	*Brasiliorchis picta*, *Maxillaria nigrescens*, *Maxillariella tenuifolia*, *M. variabilis*, *Trigonidium* cf. *turbinatum*
ar-Curcumene	*Brasiliorchis marginata*, *Chelyella jenischiana*, *Mormolyca ringens*, *Trigonidium* cf. *turbinatum*, *Xanthoxerampellia rufescens*
Aromadendrene	*Brasiliorchis gracilis*, *B. marginata*, *B. picta*, *Chelyella jenischiana*, *Heterotaxis discolor*, *Maxillaria nigrescens*, *Maxillariella tenuifolia*, *M. variabilis*, *Mormolyca ringens*, *Trigonidium* cf. *Turbinatum*, *Xanthoxerampellia rufescens*
Calarene	*Maxillariella tenuifolia*
Caryophylladienol II	*Maxillariella tenuifolia*
Caryophyllene oxide	*Brasiliorchis picta*, *Maxillaria nigrescens*, *Maxillariella tenuifolia*, *M. variabilis*
Caryophyllene	*Brasiliorchis gracilis*, *B. marginata*, *B. picta*, *Chelyella jenischiana*, *Maxillaria nigrescens*, *M. splendens*, *Maxillariella tenuifolia*, *M. variabilis*
epi-Cubebol	*Maxillariella tenuifolia*
α-Copaene	*Brasiliorchis gracilis*, *B. marginata*, *B. picta*, *Chelyella jenischiana*, *Maxillaria nigrescens*, *Maxillariella sanguinea*, *M. tenuifolia*, *M. variabilis*, *Mormolyca ringens*, *Trigonidium* cf. *turbinatum*, *Xanthoxerampellia rufescens*
α-Humulene	*Brasiliorchis gracilis*, *B. picta*, *Maxillaria nigrescens*, *Maxillariella tenuifolia*, *M. variabilis*
β-Elemene	*Brasiliorchis gracilis*, *B. marginata*, *B. picta*, *Chelyella jenischiana*, *Maxillaria nigrescens*, *Maxillariela tenuifolia*, *M. variabilis*, *Trigonidium* cf. *turbinatum*, *Xanthoxerampellia rufescens*
β-Gurjunene	*Brasiliorchis gracilis*, *B. marginata*, *Chelyella jenischiana*
β-Myrcene	*Brasiliorchis picta*, *Heterotaxis discolor*, *Maxillaria nigrescens*, *Maxillariella tenuifolia*, *M. variabilis*, *Xanthoxerampellia rufescens*
δ-Cadinene	*Brasiliorchis gracilis*, *B. marginata*, *B. picta*, *Chelyella jenischiana*, *Maxillaria nigrescens*, *Maxillariella tenuifolia*, *M. variabilis*, *Trigonidium* cf. *turbinatum*, *Xanthoxerampellia rufescens*
δ-Elemene	*Brasiliorchis gracilis*, *B. marginata*, *Chelyella jenischiana*, *Trigonidium* cf. *turbinatum*
Isocaryophyllene	*Brasiliorchis picta*, *Maxillaria nigrescens*, *Maxillariella tenuifolia*, *M. variabilis*
Erianthridin	*Chelyella densa*
Fimbriol A	*Chelyella densa*
Flavanthridin	*Maxillariella tenuifolia*
Gymnopusin	*Chelyella densa*
Nudol	*Chelyella densa*
2,5-dihydroxy-3,4-dimethoxyphenanthrene	*Chelyella densa*
2-Methoxy-4-vinylphenol	*Maxillariella sanguinea*, *M. variabilis*
Luteolin-6-C-glucoside	*Heterotaxis superflua*
Gigantol	*Chelyella densa*
Campesterol	*Maxillareilla sanguinea*
Stigmasterol	*Maxillareilla sanguinea*
2,5-di-tert-Butyl-1,4-benzoquinone	*Brasiliorchis schunkeana*, *Maxillariella vulcanica*
7,9-Di-tert-butyl-1-oxaspiro(4,5)deca-6,9-diene-2,8-dione	*Brasiliorchis schunkeana*, *Maxillariella sanguinea*, *M. tenuifolia*, *M. vulcanica*
Geranylacetone	*Brasiliorchis gracilis*, *B. marginata*, *B. picta*, *Chelyella jenischiana*, *Maxillaria nigrescens*, *Maxillariella tenuifolia*, *M. variabilis*, *Xanthoxerampellia rufescens*
Mangiferin	*Maxillariella tenuifolia*

## Data Availability

Not applicable.

## Appendix A

**Table A1:** Examples of the general occurrence of the identified compounds.

Compound	Natural Occurrence (Examples)	Family	Source of Data
Nonanal	*Citrus limon* (L.) Osbeck	Rutaceae Juss.	[161]
	*Artemisia ludoviciana* Nutt.	Asteraceae Bercht. & J. Presl	[31]
	*Brassica napus* L. var. n*apus*	Brassicaceae Burnett	[32]
	*Glycine max* (L.) Merr.	Fabaceae Lindl.	
	*Senecio laetus* Edgew.	Asteraceae Bercht. & J. Presl	
	*Haplophyllum tuberculatum* (Forssk.) A. Juss.	Rutaceae Juss.	
	*Minuartia meyeri* (Boiss.) Bornm.	Caryophyllaceae Juss.	
	*Apium graveolens* L.	Apiaceae Lindl.	
Benzaldehyde	*Prunus armeniaca* L.	Rosaceae Juss.	[162]
	*Prunus serotina* Ehrh.	Rosaceae Juss.	
	*Tagetes erecta* L.	Asteraceae Bercht. & J. Presl	[163]
	*Anacardium occidentale* L.	Anacardiaceae R. Br.	[164]
	*Prunus persica* (L.) Batsch	Rosaceae Juss.	[165]
	*Dendrobium candidum* Wall. *ex* Lindl.	Orchidaceae Juss.	[166]
	*Dendrobium chrysotoxum* Lindl.	Orchidaceae Juss.	[167]
Benzoic acid, 3-methoxy-4-hydroxy	*Vitis vinifera* L.	Vitaceae Juss.	[168]
	*Ginkgo biloba* L.	Ginkgoaceae Engl.	[169]
	*Camellia sinensis* (L.) Kuntze	Theaceae Mirb.	[170]
	*Arachis hypogaea* L.	Fabaceae Lindl.	[36]
	*Hovenia dulcis* Thunb.	Rhamnaceae Juss.	[35]
	*Zizyphus mauritiana* Lam.	Rhamnaceae Juss.	[171]
	*Angelica sinensis* (Oliv.) Diels	Apiaceae Lindl.	[37]
	*Lentinula edodes* (Berk.) Pegler (fungus)	Omphalotaceae Besl & Bresinsky	[38]
Benzoic acid, 4-ethoxy-, ethyl ester	*Curculigo orchioides* Gaertn.	Hypoxidaceae R. Br.	[172]
	*Sesuvium portulacastrum* (L.) L.	Aizoaceae Martinov	[40]
	*Salix caprea* L.	Salicaceae Mirb.	[41]
Butylated hydroxytoluene	*Betula platyphylla* var. *japonica* (Miq.) Hara	Betulaceae Gray	[173]
Cinnamic acid	*Alnus firma* Siebold & Zucc.	Betulaceae Gray	[174]
	*Litchi chinesis* Sonn.	Sapindaceae Juss.	[175]
	*Myroxylon balsamum* var. *pereirae* (Royle) Harms	Fabaceae Lindl.	[176]
	*Syzygium alternifolium* (Wight) Walp.	Myrtaceae Juss.	[177]
	*Pinus densiflora* Siebold & Zucc.	Pinaceae Spreng. *ex* Rudolphi	[47]
	*Pinus thunbergii* Lamb.	Pinaceae Spreng. *ex* Rudolphi	
	*Pinus rigida* Mill.	Pinaceae Spreng. *ex* Rudolphi	
	*Vanilla planifolia* Andrews	Orchidaceae Juss.	[178]
Cinnamic acid, 4-hydroxy-3-methoxy	*Angelica sinensis* (Oliv.) Diels	Apiaceae Lindl.	[49]
	*Cimicifuga heracleifolia* Kom.	Ranunculaceae Juss.	
	*Ligusticum chuanxiong* S.H. Qiu, Y.Q. Zeng, K.Y. Pan, Y.C. Tang & J.M. Xu	Apiaceae Lindl.	
Indole	*Cycnoches loddigesii* Lindl.	Orchidaceae Juss.	[179]
	*Gongora cassidea* Rchb. f.,	Orchidaceae Juss.	
	*Gongora quinquenervis Ruiz & Pav.*	Orchidaceae Juss.	
	*Gongora tricolor* (Lindl.) Rchb. f.	Orchidaceae Juss.	
	*Stanhopea candida* Barb. Rodr.	Orchidaceae Juss.	
	*Stanhopea* aff. *impressa* Rolfe	Orchidaceae Juss.	
	*Stanhopea tigrina* Bateman *ex* Lindl.	Orchidaceae Juss.	
p-Anisaldehyde	*Foeniculum vulgare* subsp. *vulgare* var. *dulce* (Mill.) Thellung	Apiaceae Lindl.	[180]
	*Pimpinella anisum* L.	Apiaceae Lindl.	[52]
	*Mangifera indica* L.	Anacardiaceae R. Br.	[181]
	*Allium sativum* L.	Amaryllidaceae J. St.-Hil.	[53]
	*Cuminum cyminum* L.	Apiaceae Lindl.	
	*Foeniculum vulgare* Mill.	Apiaceae Lindl.	
Azelaic acid	*Triticum* L. spp.	Poaceae Barnhart	[182]
	*Oryza sativa* L.	Poaceae Barnhart	
	*Hordeum vulgare* L.	Poaceae Barnhart	
	*Malassezia furfur* (C.P. Robin) Baill.	Malasseziaceae Denchev & R.T. Moore	
Nonanoic acid	*Ophrys sphegodes* Mill.	Orchidaceae Juss.	[183]
	*Ophrys fusca* group	Orchidaceae Juss.	[184]
	*Hibiscus syriacus* L.	Malvaceae Juss.	[58]
	*Anacamptis pyramidalis* (L.) Rich.	Orchidaceae Juss.	[185]
	*Serapias vomeracea* (Burm. f.) Briq.	Orchidaceae Juss.	
	*Dactylorhiza fuchsii* (Druce) Soó	Orchidaceae Juss.	[186]
	*Dactylorhiza incarnata* var. *incarnata*	Orchidaceae Juss.	
	*Dactylorhiza incarnata* var. *ochroleuca* Jagiello & Kuusk	Orchidaceae Juss.	
	*Dactylorhiza majalis* (Rchb. f.) P.F. Hunt & Summerh.	Orchidaceae Juss.	
	*Orchis provincialis* Balb.	Orchidaceae Juss.	[187]
	*Orchis × fallax*	Orchidaceae Juss.	
Octanoic acid	*Vitex mollis* Kunth	Lamiaceae Martinov	[62]
	*Cocos nucifera* L.	Arecaceae Bercht. & J. Presl	
Suberic acid	*Hibiscus syriacus* L.	Malvaceae Juss.	[63]
	*Vernonia galamensis* (Cass.) Less.	Asteraceae Bercht. & J. Presl	
Oleic acid	*Clusia burchellii* Engl.	Clusiaceae Lindl.	[188]
	*Clusia spiritu-sanctensis* G. Mariz & B. Weinb.	Clusiaceae Lindl.	
	*Laurus nobilis* L.	Lauraceae Juss.	[189]
	*Ophrys exaltata* Ten.	Orchidaceae Juss.	[190]
	*Rosa damascena* Mill.	Rosaceae Juss.	[40]
	*Sesuvium portulacastrum* L.	Aizoaceae Martinov	
Heptadecanoic acid	*Diplotaxis harra* Forsk.	Brassicaceae Burnett	[191]
	*Erucaria microcarpa* Boiss.	Brassicaceae Burnett	
	*Populus tremula* L.	Salicaceae Mirb.	[192]
Hexadecanoic acid	*Kigelia pinnata* (Jacq.) DC.	Bignoniaceae Juss.	[69]
	*Turbinaria ornata* (Turner) J.Agardh (algae)	Sargassaceae Kützing	[70]
Tetradecanoic acid	*Cucumis melo* L.	Cucurbitaceae Juss.	[193]
	*Cistus creticus* L.	Cistaceae Juss.	[194]
	*Elaeis guineensis* Jacq.	Arecaceae Bercht. & J. Presl	[195]
Octadecanoic acid, methyl ester	*Cymbopogon nardus* (L.) Rendle	Poaceae Barnhart	[72]
4,8,8-Trimethyl-2-methylene-4-vinylbicyclo[5.2.0]nonane	*Elsholtzia blanda* (Benth.) Benth.	Lamiaceae Martinov	[73]
	*Elsholtzia bodinieri* Vaniot	Lamiaceae Martinov	
	*Elsholtzia densa* Benth.	Lamiaceae Martinov	
	*Elsholtzia communis* (Collett & Hemsl.) Diels	Lamiaceae Martinov	
	*Mosla chinensis* Maxim.	Lamiaceae Martinov	
	*Mosla dianthera* (Buch.-Ham. *ex* Roxb.) Maxim.	Lamiaceae Martinov	
	*Mosla cavaleriei* H. Lév.	Lamiaceae Martinov	
	*Mosla scabra* (Thunb.) C.Y. Wu & H.W. Li	Lamiaceae Martinov	
Heptacosane	*Euphorbia intisy* Drake	Euphorbiaceae Juss.	[74]
2-Pentadecanone	*Pilocarpus microphyllus* Stapf *ex* Wardlew.	Rutaceae Juss.	[196]
	*Marantodes pumilum* (Blume) Kuntze	Primulaceae Batsch	[75]
2-Undecanone	*Ruta chalepensis* L.	Rutaceae Juss.	[78]
	*Zanthoxylum molle* Rehder	Rutaceae Juss.	[79]
	*Houttuynia cordata* Thunb.	Saururaceae Rich. *ex* T. Lestib.	[81]
			[82]
	*Lactobacillus plantarum* (Orla-Jensen 1919) Zheng et al. 2020	Lactobacillaceae Winslow et al. 1917	[83]
4-Terpineol	*Melaleuca alternifolia* (Maiden & Betche) Cheel	Myrtaceae Juss.	[85]
	*Juniperus communis* L.	Cupressaceae Gray	[197]
cis-β-Ocimene	*Orchis mascula* L.	Orchidaceae Juss.	[198]
	*Solanum lycopersicum* L.	Solanaceae Juss.	[199]
	*Magnolia kwangsiensis* Figlar & Noot.	Magnoliaceae Juss.	[200]
Limonene	*Citrus medica* L.	Rutaceae Juss.	[201]
	*Thymus vulgaris* L.	Lamiaceae Martinov	
	*Citrus* L. spp.	Rutaceae Juss.	[202]
	*Cannabis sativa* L.	Cannabaceae Martinov	[203]
Eucalyptol	*Coriandrum sativum* L.	Apiaceae Lindl.	[201]
	*Origanum vulgare* L.	Lamiaceae Martinov	
	*Rosmarinus officinalis* L.	Lamiaceae Martinov	
	*Thymus vulgaris* L.	Lamiaceae Martinov	
	*Zingiber officinale* Roscoe	Zingiberaceae Martinov	
	*Stanhopea anfracta* Rolfe	Orchidaceae Juss.	[28]
	*Croton rhamnifolioides* Pax & K. Hoffm.	Euphorbiaceae Juss.	[204]
	*Eucalyptus* L’Hér.	Myrtaceae Juss.	[97]
	*Salvia lavandulifolia* Vahl.	Lamiaceae Martinov	
	*Melaleuca quinquenervia* (Cav.) S.T. Blake	Myrtaceae Juss.	
	*Elsholtzia blanda* (Benth.) Benth.	Lamiaceae Martinov	[73]
γ-Terpinene	*Lippia gracilis* Schauer	Verbenaceae J. St.-Hil.	[205]
	*Melaleuca alternifolia* (Maiden & Betche) Cheel	Myrtaceae Juss.	[206]
	*Lippia multiflora* Moldenke	Verbenaceae J. St.-Hil.	[207]
Linalool	*Salvia sclarea* L.	Lamiaceae Martinov	[100]
	*Salvia desoleana* Atzei & Picci	Lamiaceae Martinov	
p-Cymene	*Thymus vulgaris* L.	Lamiaceae Martinov	[208]
	*Protium* Burm. f. spp.	Burseraceae Kunth	[102]
	*Artemisia* L. spp.	Asteraceae Bercht. & J. Presl	[103]
	*Eucalyptus* L’Hér. spp.	Myrtaceae Juss.	
	*Ocimum* L. spp.	Lamiaceae Martinov	
	*Protium* Burm. f. spp.	Burseraceae Kunth	
α-Pinene	*Salvia officinalis* L.	Lamiaceae Martinov	[209]
	*Pinus* L. spp.	Pinaceae Spreng. *ex* Rudolphi	[105]
	*Cinnamomum verum* J. Presl	Lauraceae Juss.	[106]
	*Coriandrum sativum* L.	Apiaceae Lindl.	
	*Cuminum cyminum* L.	Apiaceae Lindl.	
	*Juniperus communis* L.	Cupressaceae Gray	
	*Lavandula stoechas* L.	Lamiaceae Martinov	
	*Melaleuca alternifolia* Cheel.	Myrtaceae Juss.	
	*Ocimum menthaefolium* Benth.	Lamiaceae Martinov	
	*Rosmarinus officinalis* L.	Lamiaceae Martinov	
α-Terpineol	*Salvia officinalis* L.	Lamiaceae Martinov	[209]
	*Origanum vulgare* L.	Lamiaceae Martinov	[107]
	*Ocimum canum* Sims	Lamiaceae Martinov	
	*Artemisia rupestris* L.	Asteraceae Bercht. & J. Presl	[108]
	*Juniperus communis* L.	Cupressaceae Gray	
	*Myristica fragrans* Houtt.	Myristicaceae R. Br.	
	*Salvia sclarea* L.	Lamiaceae Martinov	
β-Pinene	*Salvia officinalis* L.	Lamiaceae Martinov	[209]
	*Pinus* L. spp.	Pinaceae Spreng. *ex* Rudolphi	[105]
	*Cinnamomum verum* J. Presl	Lauraceae Juss.	[106]
	*Coriandrum sativum* L.	Apiaceae Lindl.	
	*Cuminum cyminum* L.	Apiaceae Lindl.	
	*Juniperus communis* L.	Cupressaceae Gray	
	*Lavandula stoechas* L.	Lamiaceae Martinov	
	*Melaleuca alternifolia* Cheel.	Myrtaceae Juss.	
	*Ocimum menthaefolium* Benth.	Lamiaceae Martinov	
	*Rosmarinus officinalis* L.	Lamiaceae Martinov	
ar-Curcumene	*Curcuma aromatica* Salisb.	Zingiberaceae Martinov	[210]
	*Curcuma xanthorrhiza* Roxb.	Zingiberaceae Martinov	
	*Zingiber officinale* Roscoe	Zingiberaceae Martinov	[109]
	*Pogostemon cablin* (Blanco) Benth.	Lamiaceae Martinov	[211]
Aromadendrene	*Eucalyptus globulus* Labill.	Myrtaceae Juss.	[95]
Calarene	*Kadsura heteroclita* (Roxb.) Craib	Schisandraceae Blume	[110]
Caryophylladienol II	*Achillea cretica* L.	Asteraceae Bercht. & J. Presl	[111]
	*Salvia verticillata* subsp. *amasiaca* (Freyn & Bornm.) Bornm.	Lamiaceae Martinov	[114]
Caryophyllene oxide	*Pilocarpus microphyllus* Stapf *ex* Wardlew.	Rutaceae Juss.	[196]
	*Annona squamosa* L.	Annonaceae Juss.	[112]
	*Salvia verticillata* subsp. *amasiaca* (Freyn & Bornm.) Bornm.	Lamiaceae Martinov	[114]
Caryophyllene	*Pilocarpus microphyllus* Stapf *ex* Wardlew.	Rutaceae Juss.	[196]
	*Piper cubeba* L.	Piperaceae Giseke	[212]
	*Cannabis sativa* L.	Cannabaceae Martinov	[113]
	*Cinnamomum* Scheffer spp.	Lauraceae Juss.	
	*Lavandula angustifolia* Mill.	Lamiaceae Martinov	
	*Ocimum* L. spp.	Lamiaceae Martinov	
	*Origanum vulgare* L.	Lamiaceae Martinov	
	*Piper nigrum* L.	Piperaceae Giseke	
	*Rosmarinus officinalis* L.	Lamiaceae Martinov	
	*Syzygium aromaticum* (L.) Merr. & L.M. Perry	Myrtaceae Juss.	
epi-Cubebol	*Piper cubeba* L.	Piperaceae Giseke	[212]
	*Cryptomeria japonica* (Thunb. *ex* L. f.) D. Don	Cupressaceae Gray	[119]
	*Chromolaena odorata* (L.) R. M. King & H. Rob	Asteraceae Bercht. & J. Presl	[213]
α-Copaene	*Annona reticulate* L.	Annonaceae Juss.	[120]
	*Cedrelopsis grevei* Baill.	Rutaceae Juss.	
	*Ceratitis capitata* (Wiedemann, 1824) (medfly)	Tephritidae Newman	
	*Xylopia laevigata* (Mart.) R.E. Fr.	Annonaceae Juss.	
α-Humulene	*Abies balsamea* (L.) Mill.	Pinaceae Spreng. *ex* Rudolphi	[121]
	*Cordia verbenacea* DC.	Cordiaceae R. Br. *ex* Dumort.	[122]
	*Piper aduncum* L.	Piperaceae Giseke	
	*Polyalthia cerasoides* (Roxb.) Benth. & Hook. f. *ex* Bedd.	Annonaceae Juss.	
	*Salvia officinalis* L.	Lamiaceae Martinov	[209]
	*Mentha spicata* L.	Lamiaceae Martinov	[123]
	Zingiberaceae Martinov	Zingiberaceae Martinov	
β-Elemene	*Piper cubeba* L.	Piperaceae Giseke	[212]
	*Curcuma* L. spp.	Zingiberaceae Martinov	[214]
β-Gurjunene	*Dipterocarpus alatus* Roxb.	Dipterocarpaceae Blume	[127]
β-Myrcene	*Cymbopogon citratus* (DC.) Stapf	Poaceae Barnhart	[128]
	*Humulus lupulus* L.	Cannabaceae Martinov	
	*Laurus* L. spp.	Lauraceae Juss.	
	*Verbena* L. spp.	Verbenaceae J. St.-Hil.	
δ-Cadinene	*Xylopia laevigata* (Mart.) R.E. Fr.	Annonaceae Juss.	[102]
	*Uncaria rhynchophylla* (Miq.) Miq. *ex* Havil.	Rubiaceae Juss.	[215]
	*Piper trioicum* Roxb.	Piperaceae Giseke	[216]
	*Marrubium friwaldskyanum* Boiss.	Lamiaceae Martinov	[217]
	*Neuropeltis acuminata* (P.Beauv.) Benth.	Convolvulaceae Juss.	[218]
	*Centaurea cyanus* L.	Asteraceae Bercht. & J. Presl	[219]
δ-Elemene	*Pelargonium endlicherianum* Fenzl	Geraniaceae Juss.	[220]
	*Achillea millefolium* L.	Asteraceae Bercht. & J. Presl	[221]
	*Commiphora holtziana* Engl.	Burseraceae Kunth	[222]
Isocaryophyllene	*Couroupita guianensis* Aubl.	Lecythidaceae A. Rich.	[223]
	*Ficus carica* L.	Moraceae Gaudich.	[224]
	*Teucrium marum* L.	Lamiaceae Martinov	[131]
Erianthridin	*Eria convallarioides* Lindl.	Orchidaceae Juss.	[225]
	*Dendrobium plicatile* Lindl.	Orchidaceae Juss.	[226]
Fimbriol A	*Scaphyglottis livida* (Lindl.) Schltr.	Orchidaceae Juss.	[133]
Gymnopusin	*Bulbophyllum gymnopus* Hook. f.	Orchidaceae Juss.	[227]
Nudol	*Eria convallarioides* Lindl.	Orchidaceae Juss.	[225]
	*Dendrobium nobile* Lindl.	Orchidaceae Juss.	[140]
2,5-Dihydroxy-3,4-dimethoxyphenanthrene	*Dendrobium candidum* Wall. *ex* Lindl.	Orchidaceae Juss.	[228]
2-Methoxy-4-vinylphenol	*Fagopyrum esculentum* Moench	Polygonaceae Juss.	[142]
	*Malus* Mill. spp.	Rosaceae Juss.	
	*Arachis hypogaea* L.	Fabaceae Lindl.	
	*Syzygium aromaticum* (L.) Merr. & L.M.Perry	Myrtaceae Juss.	
Luteolin-6-C-glucoside	*Arum palaestinum* Boiss.	Araceae Juss.	[143]
	*Bryonia* L. spp.	Cucurbitaceae Juss.	[144]
	*Gentiana* L. spp.	Gentianaceae Juss.	
	*Piptadenia* Benth. spp.	Fabaceae Lindl.	
	*Tamarindus* L. spp.	Fabaceae Lindl.	
	*Mauritia flexuosa* L. f.	Arecaceae Bercht. & J. Presl	[98]
	*Achillea oligocephala* DC	Asteraceae Bercht. & J. Presl	[145]
	*Callisia fragrans* (Lindl.) Woodson	Commelinaceae Mirb.	[229]
Gigantol	*Cymbidium goeringii* (Rchb. f.) Rchb. f.	Orchidaceae Juss.	[146]
Campesterol	*Chrysanthemum coronarium* L.	Asteraceae Bercht. & J. Presl	[149]
	*Argania spinosa* (L.) Skeels	Sapotaceae Juss.	[230]
	*Wrightia tinctoria* (Roxb.) R. Br.	Apocynaceae Juss.	[231]
Stigmasterol	*Butea monosperma* (Lam.) Taub.	Fabaceae Lindl.	[150]
2,5-Di-tert-Butyl-1,4-benzoquinone	*Bacillus Cohn* spp. (bacteria)	Bacillaceae Garrity et al. 2001	[152]
	*Streptomyces* sp. VITVSK1 (bacteria)	Streptomycetaceae Waksman and Henrici 1943	
	*Bulbophyllum echinolabium* J.J. Sm.	Orchidaceae Juss.	[232]
7,9-Di-tert-butyl-1-oxaspiro(4,5)deca-6,9-diene-2,8-dione	*Mangifera indica* L.	Anacardiaceae R. Br.	[233]
	*Gmelina asiatica* Linn	Lamiaceae Martinov	[234]
	*Cordia sebestena* L.	Cordiaceae R. Br. *ex* Dumort.	[235]
	*Cyperus rotundus* L.	Cyperaceae Juss.	[236]
	*Cyathea nilgirensis* Holttum	Cyatheaceae Kaulf.	[237]
	*Cuscuta reflexa* Roxb.	Convolvulaceae Juss.	[238]
	*Bulbophyllum echinolabium* J.J. Sm.	Orchidaceae Juss.	[232]
Geranyl- acetone	*Lycopersicon esculentum* Mill.	Solanaceae Juss.	[239]
	*Conyza bonariensis* L.	Asteraceae Bercht. & J. Presl	[158]
	*Equisetum arvense* L.	Equisetaceae Michx. *ex* DC.	
	*Ononis natrix* L.	Fabaceae Lindl.	
Mangiferin	*Mangifera indica* L.	Anacardiaceae R. Br.	[160]
